# Viscoelasticity enhances collective motion of bacteria

**DOI:** 10.1093/pnasnexus/pgad291

**Published:** 2023-09-06

**Authors:** Wentian Liao, Igor S Aranson

**Affiliations:** Department of Biomedical Engineering, Pennsylvania State University, University Park, PA 16802, USA; Department of Biomedical Engineering, Pennsylvania State University, University Park, PA 16802, USA

**Keywords:** biological fluids, mucus, viscoelasticity, bacterial motility

## Abstract

Bacteria form human and animal microbiota. They are the leading causes of many infections and constitute an important class of active matter. Concentrated bacterial suspensions exhibit large-scale turbulent-like locomotion and swarming. While the collective behavior of bacteria in Newtonian fluids is relatively well understood, many fundamental questions remain open for complex fluids. Here, we report on the collective bacterial motion in a representative biological non-Newtonian viscoelastic environment exemplified by mucus. Experiments are performed with synthetic porcine gastric mucus, natural cow cervical mucus, and a Newtonian-like polymer solution. We have found that an increase in mucin concentration and, correspondingly, an increase in the suspension’s elasticity monotonously increases the length scale of collective bacterial locomotion. On the contrary, this length remains practically unchanged in Newtonian polymer solution in a wide range of concentrations. The experimental observations are supported by computational modeling. Our results provide insight into how viscoelasticity affects the spatiotemporal organization of bacterial active matter. They also expand our understanding of bacterial colonization of mucosal surfaces and the onset of antibiotic resistance due to swarming.

**Significance Statement**

Mucus, a gel-like viscoelastic substance, is essential for many biological functions. Mucus lines the surfaces of cells and tissues. It is permeable to oxygen and nutrients and protects against pathogens such as bacteria, fungi, and viruses. Understanding bacterial motility in mucus-like fluids provides insights into bacteria-born infections, including sexually transmitted and gastric diseases. This work demonstrates that mucus viscoelasticity enhances bacterial organization, leading to the emergence of coherently moving bacterial groups. The results shed light on how viscoelasticity controls the spatiotemporal organization of bacterial communities and provide insight into controlling and preventing bacterial invasion of mucosal surfaces.

## Introduction

Bacteria are the most abundant species on Earth. They compose human and animal microbiota and are sources of many infectious diseases (1). Suspension of motile bacteria is an important class of active matter: nonequilibrium systems transducing energy from the environment into mechanical motion. Concentrated bacterial suspensions often exhibit large-scale turbulent-like motion (so-called bacterial turbulence) (2, 3). While significant knowledge is accumulated on the collective dynamics of bacterial suspensions in Newtonian liquids, complex fluids are mostly “terra incognita” (4).

Bacterial habitats are not limited to Newtonian fluids such as water. Bacteria thrive in non-Newtonian environments (4) exemplified by lyotropic liquid crystals (5, 6), mucus (7, 8), DNA solutions (9, 10), blood (11), saliva (12), biofilm matrices (13), or mammalian extracellular matrices (14). Unlike the simple viscous response in a Newtonian liquid, the non-Newtonian rheology of biological viscoelastic fluid can drastically alter the bacterial collective motion (15, 16). However, there is no consensus even on the level of individual motility of microorganisms in viscoelastic fluids. For example, some studies predicted that viscoelasticity hinders self-propulsion (17, 18). On the contrary, other investigations suggested that the microswimmers propel faster or slower in the viscoelastic fluid but slower in a shear-thinning fluid (19, 20). Alternatively, computational modeling (21) predicted increased bacterial swimming speed up to 60% in polymer solutions due to a nonuniform distribution of polymer molecules around a bacterium. Also, computational studies revealed that viscoelasiticity significantly affect collective motion of bacteria-like rod-shaped swimmers (pushers) but has a small effect on a suspension of algae-like swimmers (pullers) (15). Thus, examination of collective and individual dynamics of bacteria in viscoelastic fluids may produce new insights into active matter in complex environments.

Among biological fluids, mucus, a gel-like viscoelastic substance lining the exposed organism tract, is a crucial habit of bacterial communities. The dominant glycan protein, mucin, forms a mesh network and hence endows mucus elasticity (22). Mucus demonstrates non-Newtonian dynamics: shear-thinning, stress relaxation, yielding behavior (23), and even some liquid crystallinity (7, 24). Most studies focus on the penetration of individual bacteria or sperm across mucus. The individual motility of bacteria such as *Helibactor pylori*, *Escherichia coli*, and *Bacillus subtilis* in mucus has been studied to better understand both the defensive mechanism of mucus and the invasion strategy of bacteria (7, 8, 25). The motility of individual sperm in cervical mucus has also been investigated to understand the mechanisms of infertility (26). However, the collective states of concentrated bacteria in mucus, which is relevant in the context of emerging antibiotic resistance (27, 28) and colonization of mucosal surfaces in intestinal infection (29), is mostly unexplored.

Here, we examine the collective motion of concentrated bacteria *Bacillus subtilis* in synthetic porcine gastric mucus and natural cow cervical mucus. We contrasted the collective behavior in viscoelastic mucus with essentially Newtonian polyvinylpyrrolidone (PVP360) polymer solution at a wide range of concentrations. We have found that the velocity correlation length of bacterial collective locomotion increases monotonously with the increase of mucin concentration in both synthetic mucus and natural mucus. The elevation of mucin concentration increases both storage and loss modules which is consistent with a previous observation (23). On the contrary, the correlation length of bacterial motion in the PVP360 solution remained practically unchanged regardless of PVP360 concentration, consistent with the correlation length predictions for Newtonian fluid (30). The study implies that the spatial correlation of bacterial active matter is primarily affected by fluid elasticity rather than viscosity. The experimental results are supported by the computational analysis of phenomenological model for bacterial collective motion in viscoelastic fluids. The result sheds light on manipulating active matter by modifying the elasticity of the suspending fluid.

## Results

**Fig. 1.**
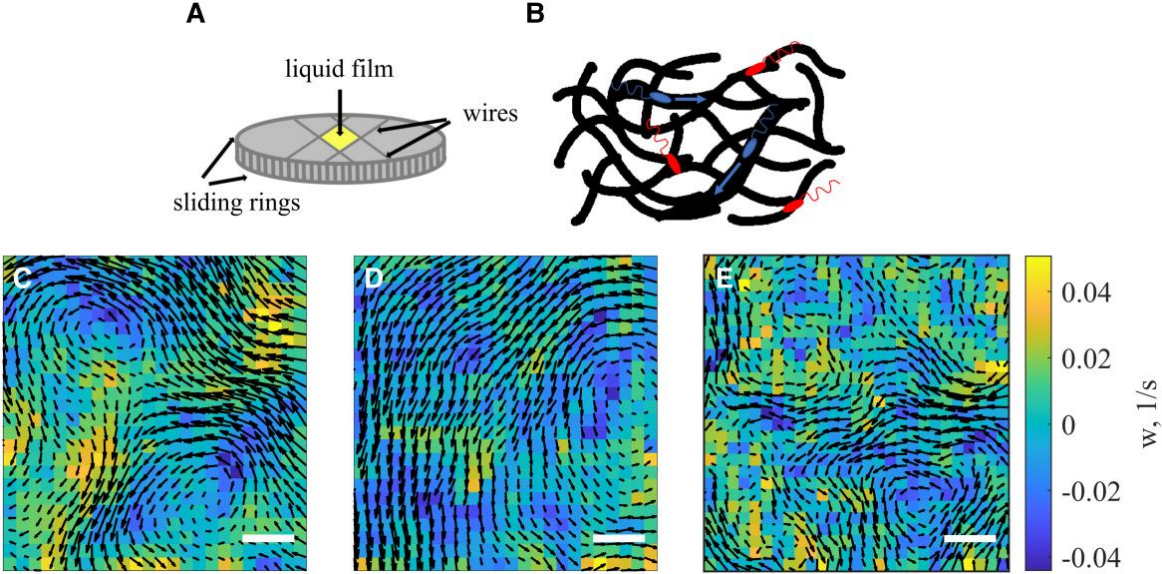
Instant flow patterns of bacterial collective motion in mucus and PVP360. A) Schematics of the experimental setup. A free-standing film containing bacterial suspension in mucus is stretched between 4 movable wires. B) A schematic representation of motile bacteria (blue bodies with flagella) swimming in tunnels formed by mucin polymers (black tubes). The blue (brighter in grey-scale image representation) arrow indicates the swimming direction of mobile bacteria. The trapped bacteria are shown in red. C–E) Select frames illustrating instant flow patterns at the bottom of the film for different concentrations of mucin/PVP360; black arrows depict the direction and magnitude of bacterial flow, and colors show the vorticity *w* of bacterial flow. C) Instant flow pattern in 50 mg/mL mucin solution. D) Instant flow pattern in 200 mg/mL mucin solution. E) Instant flow pattern in 125 mg/mL PVP360 solution. The scale bar in all figures is 80 μm.

Experiments were performed with motile aerobic bacteria *Bacillus subtilis*, strain DK400. The bacteria were grown in a Terrific Broth (TB) medium (Sigma T5574) and then centrifuged to obtain a condensed bacterial pellet. The pellet was diluted in fresh TB/synthetic mucus/natural mucus/PVP360 solutions to reach concentrations of the order $1-3\times 10^{10}$ cells/cm${}^{3}$. Then, the bacterial suspension was pipetted into a free-standing film to ensure sufficient oxygen supply for the respiration of bacteria, Fig. 1A. A similar setup was used for the experiments on bacterial locomotion in Newtonian fluids (30). The bright-field or fluorescent images were recorded at the rate of about 33 frames per second and then processed with PIVlab (31) and custom MATLAB script.

**Fig. 2.**
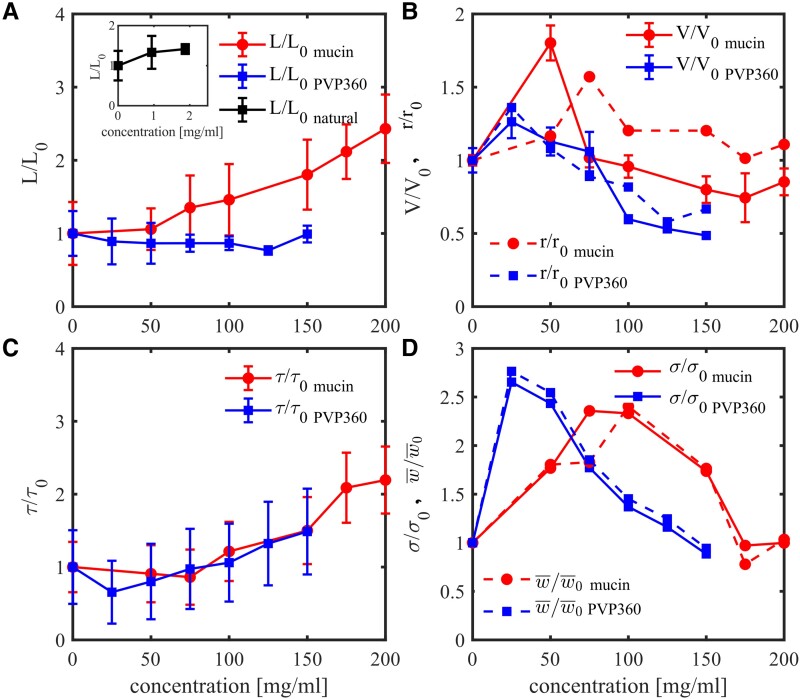
Properties of bacterial collective motion in mucus and PVP360. A) Velocity correlation length *L* for different mucin/PVP360 concentrations. The correlation length is normalized on the correlation length $L_{0}\approx 80-100$μm in pure TB. Inset: Correlation length *L* for different natural mucus concentrations. B) The root mean square (rms) velocity of collective motion *V* for different mucin/PVP360 concentrations. The velocity is normalized on velocity $V_{0}\approx 43$μm/s in pure TB. The rms velocity is compared with the normalized correlation velocity ($r/r_{0}$, $r_{0}=L_{0}/\tau_{0}$) defined by normalized correlation length divided by normalized correlation time. C) Velocity correlation time τ for different mucin/PVP360 concentrations. The correlation time is normalized on the correlation time $\tau_{0}\approx 3$ s in pure TB. D) The standard deviation of the vorticity probability distribution σ and the mean vorticity absolute value $\bar{w}$ for different mucin/PVP360 concentrations. Solid blue squares and red circles (brighter in grey-scale image representation) represent PVP360 and mucin concentrations, respectively. In all panels, the symbols depict the measured values, lines are the guides for the eye. Error bars are the standard deviations.

Synthetic mucus, fabricated by mixing purified porcine stomach mucin powder (M1778 Sigma-Aldridge) with TB, was used in the experiment. The mucin solution’s most profound feature is the deformable polymer network which endows mucus with viscoelasticity, Fig. 1B. Synthetic mucin solution preserves most of the rheological properties of fresh mucus that is hard to acquire consistently in large quantities (Supplementary Fig. S1). Natural mucus was collected directly from the cervical cow tract and diluted with broth immediately. Two dilutions with mucin concentrations of 0.9375 and 1.875 mg/mL were used for experiments in the inset to Fig. 2A; see Methods for determining mucin concentration. For simplification, all mucin solutions mentioned in the following text represents synthetic mucus unless specified explicitly. Control experiments were conducted with a Newtonian suspension of PVP360 Sigma-Aldridge. The measurements were performed in a wide range of mucin and PVP360 concentrations.

Mucus microrheological properties are summarized in Discussions and Supplementary Fig. S1. See also Methods for describing the technique. Overall, mucus exhibits viscoelastic behavior with the effective elastic constant *E* monotonously increasing with the increase in the mucin concentration. On the contrary, the PVP360 solution shows a negligible elastic response in the entire range of the polymer concentrations. A similar monotonous increase was also observed in macrorheological measurements; see Supplementary Fig. S1. However, the results have a significant scatter due to the difficulty of uniformly loading of the sample at high mucin concentrations.

To examine the effect of viscoelasticity on collective behavior, we recorded the image sequences of bacterial motion in viscoelastic mucus and viscous Newtonian PVP360 solutions. We determined the velocity field from the particle-image velocimetry (PIV) using the PIVlab software. Select results for the velocity fields in synthetic mucus and PVP360 solutions are shown in Fig. 1C–E. Visual comparison of the velocity field snapshots Fig. 1C and D (mucin) and Fig. 1E (PVP360) indicates a drastically different response of the bacterial collective motion on the increase in the mucin/PVP360 concentration. In the concentrated mucin solution (Fig. 1D), the spatial scale of collective motion increases by a factor of 2–3 compared to the dilute case (Fig. 1C). In the PVP360 solution, the correlation length remains practically unchanged regardless of the polymer concentration (Fig. 1E). See also Supplementary Videos 1–4.

**Fig. 3.**
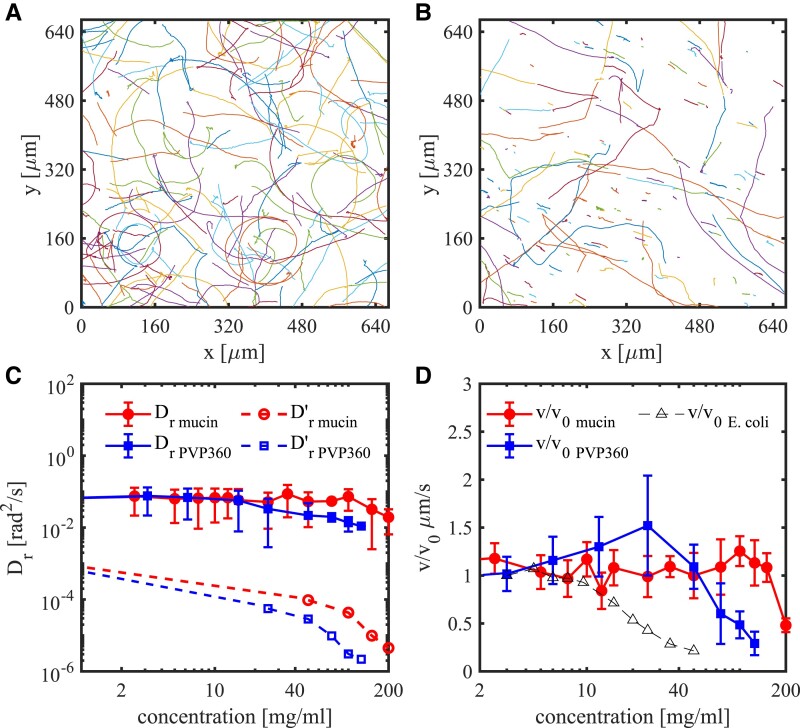
Characterization of individual bacterial dynamics. A) Trajectories of individual bacteria in pure TB. B) Trajectories of individual bacteria in 100 mg/mL mucin solution. C) The rotational diffusion coefficient of individual bacteria in mucin/PVP360 solutions at different concentrations where the solid blue line with solid squares and solid red line (brighter in grey-scale image representation) with solid circles represents measured value in PVP360 and mucin solutions, respectively. The blue dashed line with empty squares and the red dashed line with empty circles represent expected values for thermal rotational diffusion in PVP360 and mucin solution, respectively. D) Individual bacterial speeds (v) scaled by the speed in pure TB ($v_{0}$) in mucin/PVP360 solutions at different concentrations where the solid blue line with solid squares and solid red line with solid circles represents PVP360 and mucin solution, respectively. Individual bacterial speeds from Ref. (34) in PVP360 polymer solutions are shown with empty black triangles. In all panels, the symbols depict the measured values; lines are the guides for the eye. Error bars are the standard deviations.

**Fig. 4.**
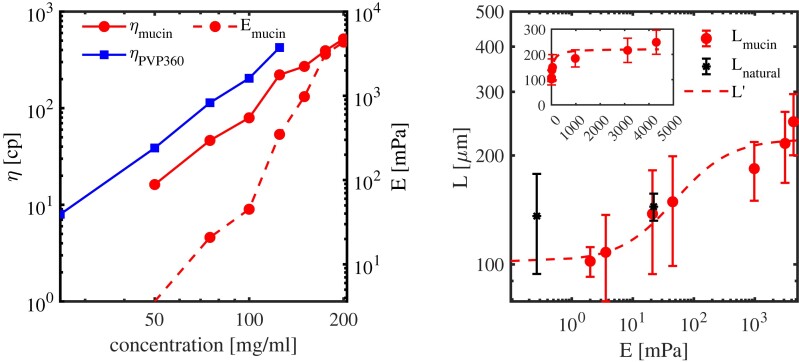
Dependence of collective motion on the elastic properties of mucus. A) Microrheological measurements of mucin/PVP360 solutions at different concentrations. Red circles (brighter in grey-scale image representation) correspond to the effective viscosity η (solid line) and elasticity *E* (dashed line) of mucin solutions. Blue solid line with solid squares corresponds to the effective viscosity η of PVP360 solutions. PVP360 is effectively viscous Newtonian fluid so no detectable elasticity is observed. The viscosity is defined as $G^{\prime \prime }(\omega )/\omega$ at ω=1 Hz, where $G^{\prime \prime }(\omega )$ is the loss modulus at the measurement frequency ω. The elasticity *E* is defined as $G^{\prime }(\omega )$ at ω=5 Hz, where $G^{\prime }(\omega )$ is the storage modulus. B) Elasticity *E* determines the correlation length *L*. The red solid circles shows the dependence of the correlation length *L* in mucin solution. The black solid circles shows the dependence of the correlation length *L* for different natural mucus concentrations. The symbols depict the measured values. Red dashed line is the theoretical prediction, Eq. 7, $L\approx L_{0}\sqrt{\left(1+\kappa_{1}E)/(1+\kappa_{2}E\right)}$ with $E=G^{\prime }$ taken at ω=5 Hz. $\kappa_{1,2}$ are fitting parameters. Inset: The dependence of the correlation length *L* vs. mucus elasticity in linear scale.

Further characterization of collective and individual bacterial dynamics is presented in Figs. 2 and 3. First, we determined the correlations length *L* dependencies on concentration of mucin and PVP360; the main results are shown in Fig. 2A. From the experimental velocity fields, we extracted the structure factors and the radial spatial correlation functions K(r). The corresponding velocity correlation length *L* was obtained by fitting the correlation function K(r) to an exponential function K(r)∼exp(−r/L)+const. The correlation length (about 100 μm) at zero mucin concentration (pure TB) is one order magnitude higher than a typical bacteria size (about 0.8 μm in diameter and 5–7 μm in length), confirming an emergence of collective behavior. The change of the mucin content in synthetic mucus from 0 to 200 mg/mL increases the correlation length *L* by a factor of two or three, from 80–100 to 250 μm. At this concentration range, the effective (macroscopic) viscosity of mucin solution changes by two orders of magnitude associated with the increase in elasticity at a much higher rate as shown in Fig. 4A.

Similarly, the correlation length in natural cow cervical mucus increases with mucin concentration and reaches 144 μm at a mucin concentration of around 1.875 mg/mL as shown in the inset to Fig. 2A. However, reproducible experiments with natural mucus are challenging. When mucin concentration is above 1.875 mg/mL, the natural mucus network prevents the homogeneous mixing of bacterial pellets in mucus without breaking the innate mucus network. As a result, the experiments with natural mucus at a mucin concentration higher than 1.875 mg/mL are very sensitive to the preparation details and therefore there are only two data points for natural mucus in the inset to Fig. 2A. An example of bacterial collective motion in natural mucus at a concentration higher than 1.875 mg/mL (3.75 mg/mL) is shown in Supplementary Video 5 with a spatial scale of ∼200 μm, which is close to that in 200 mg/mL synthetic mucus. Overall, the correlation length *L* dependencies on mucin concentration in natural mucus exhibit a similar monotonous increase. Still, they could be different in values because the restored mucus network from synthetic mucus has smaller viscoelasticity compared with fresh, natural mucus at the same mucin concentration (32).

Compared to mucus, increasing the PVP concentration does not affect the spatial scale of collective motion, Fig. 2A. The main impact of PVP360 is slowing down the dynamics. Across the whole PVP360 concentration range, the effective (macroscopic) viscosity of suspension changes by two orders of magnitude. However, the corresponding elasticity is negligible compared to mucus (see Discussions and Fig. 4A). The results imply that fluid elasticity enhances correlation length while fluid viscosity mainly slows down the dynamics.

Figure 2B displays the root mean square (rms) velocity of collective motion *V*. At 0 mg/mL (pure TB), the rms velocity of collective motion is about 43 μm/s, consistent with the previous study (30). The rms velocity of individual bacteria in TB with a viscosity of approximately 1 cp is about 20 μm/s. The doubled swimming speed indicates the emergence of collective motion. The rms speed of bacterial flow continues to increase after 0 mg/mL and peaks at 50 mg/mL for mucus and 25 mg/mL for PVP360 solution, with a value of around 77 μm/s, and 53 μm/s, respectively.

After the peak, the speed in both fluids gradually decreases and reaches a plateau at 30 μm/s for mucin solution and 21 μm/s for PVP360 solution. In all concentrations, the speed of collective motion in PVP360 is always lower than in mucin solution. It results from the higher viscosity of PVP360 solution in the same concentration, see Discussions. A plausible explanation for the velocity increase is the suppression of the velocity fluctuations due to polymer stretching and reorientation by swimming bacteria (33). At higher concentrations (over 50 mg/mL), the increased suspension viscosity slows the bacteria down. However, compared to viscoelastic mucus, the swimming speed enhancement in viscous but otherwise Newtonian polymer liquid is modest. For comparison, we plot in Fig. 2B the correlation velocity defined r=L/τ. The correlation velocity *r* exhibits a similar nonmonotonic trend as the rms velocity *V*, although the peak is less pronounced.

We further examined the temporal velocity correlation time *τ* dependencies on concentration of mucin and PVP360. To determine *τ*, each image was partitioned into 127×127 subdomains. The correlation time was extrapolated by fitting the velocity autocorrelation function at each subdomain into the exponential dependence (as for spatial correlations). The correlation time of all subdomains was averaged to obtain the mean value. Figure 2C presents correlation time *τ* as a function of concentration. Unlike the correlation length *L* dependencies, the correlation times increase with the increase of solid fraction for both mucus and PVP360 solution at most concentrations (>25 mg/mL). This observation complies with the increase of effective viscosity of the suspension with the increase of solid fraction and, corresponding, overall slowdown of the motion (30). However, mucus and PVP360 solutions show a minor decrease in the correlation time for small concentrations, about 25 mg/mL. This decrease in the correlation time is likely related to the increase in the velocity of collective motion.

Figure 2D shows the vorticity magnitude dependencies. We use two complementary definitions of vorticity magnitudes $\sigma ,\bar{w}$ to provide a more comprehensive characterization of bacterial collective dynamics. To extract σ and $\bar{w}$ from the sequence of experimental images, we calculated the vorticity *w* from the velocity field. Vorticity magnitude $\bar{w}$ is the mean absolute value of vorticity *w*. The magnitude σ is defined as the standard deviation of the vorticity probability distribution P(w) approximated by the Gaussian law $P(w)∼\exp\;(-w^{2}/2\sigma^{2})$; see Supplementary Figs. S2–S5. As one sees from Fig. 2D, different definitions $\sigma ,\bar{w}$ exhibit similar trends. It implies that the probability distribution P(w) is close to the Gaussian one. The rms velocity *V* and the vorticity magnitudes *σ* and $\bar{w}$ exhibit similar nonmonotonic trends vs. concentration of mucin or PVP360, Fig. 2B and D: initial increase followed by a gradual decrease.

We investigated the movements of individual bacteria in synthetic mucus and PVP360 solutions to obtain additional insights into of the correlation length dependence in a viscoelastic fluid. The experiments on the individual bacterial dynamics are performed on the glass slide. Due to the interaction between bacteria and solid surface, the bacteria move on circular orbits in Newtonian fluids (35), Fig. 3A. However, in polymer solutions, the trajectories can straighten due to a viscoelastic lift force, Fig. 3B (36). This viscoelastic lift force does not necessarily affect the bacterial collective dynamics studied in a surface-free standing film. Also, as the solid fraction of mucin solution increases with concentration, the relative fraction of trapped or slowly moving bacteria increases, as displayed by the decreased number of trajectories presented in Fig. 3B, also see Supplementary Videos 6–8. The short trajectories less than 20 μm are the results of impurity in mucin solution and bacteria trapped in the mucin mesh. They have been filtered out before the calculation on velocity and rotational diffusion coefficient.

We examined individual bacteria’s rotational diffusion $D_{R}$. It was calculated by fitting the mean square angular displacement (MSAD) $\left\langle \Delta \theta^{2}\right\rangle$ of each tracked trajectory into the linear law $\langle \Delta \theta^{2}\rangle =2D_{R}t$. The MSADs of tracked trajectories in mucin/PVP360 solution are shown in Supplementary Figs. S6–S8. The black dashed line represents the average, respectively. The rotational diffusion, see Fig. 3C, shows no apparent dependence on the mucin concentration (solid red line) until ∼ 50 mg/mL, after which it decreases with viscosity. However, $D_{R}$ gradually decreases in PVP360 solutions with the concentration increase in the whole range (solid blue line). Estimates for the thermal rotational diffusion $D_{R}^{\prime }$ (red and blue dashed lines) are shown in Fig. 3C to highlight the differences in rotational diffusion in mucin/PVP360 solutions. The estimate considers the medium to be homogeneous and isotropic across all concentrations in mucin and PVP360 solutions. Thus $D_{R}^{\prime }$ should decrease inversely with the effective viscosity *η* as the red and blue dashed lines in Fig. 3C. However, the mucin solution is not homogenous neither isotropic due to the polymer network, Fig. 1B. Inside the pore space of the mesh network, the bacteria can swim as they do in the solvent, giving a similar rotational diffusion as in broth. Therefore, the tracked bacteria produce a rotational diffusion in mucin solution with a much weaker concentration dependence compared with PVP360 solution. In the PVP360 solution, where the medium is more homogeneous and isotropic for bacteria, the rotational diffusions decrease inversely with the effective viscosity, as has been reported by previous studies (37).

The speed of individual bacteria also shows different concentration dependencies in mucin and PVP360 solutions. In mucin solutions, the motile bacteria have a similar speed as in TB until ∼100 mg/mL and decreases then. The difference hints at the specific mucin solution structure. A cow cervical mucus shows a mesh network with an average pore size of around 2 μm (7). Inside the mesh network, the fluid viscosity is similar to or only slightly higher than that of water. Therefore, the motion of bacteria inside the mucus pores is similar to that in water. As the concentration of mucin increases, the mesh pore size decreases, which will trap more bacteria. Hence, a smaller number of motile bacteria is observed. However, the speed of motile bacteria is still the same since the viscosity inside the pore does not change. In PVP360 solutions, the speed initially increases until 20 mg/mL and decreases afterward. The initial increase was expected to be the influence of the small molecular weight impurity from PVP360 solutions (34). After 20 mg/mL, the speed decreases as the viscosity increases.

## Discussions

Our experimental study revealed a distinct effect of viscoelasticity: while the medium viscosity results in the overall slowdown of collective motion, the elasticity increases the correlation length of collective motion. By examining individual bacterial dynamics, one may come to the conclusion that an increase in the correlation length is an outcome of the straightening of bacterial trajectories (33, 37). However, this issue is more subtle, and the curvature reduction of individual bacteria trajectories does not necessarily translates into the increase of the collective motion scale, as shown by our study, see Fig. 1C–E. Straightening of the bacterial trajectories could be an interplay of multiple factors: suppression of run-and-tumble behavior, bundling of multiple flagella in a single tight bundle due to polymer interaction, effect of the lift force near solid surface, or alignment of bacterial bodies with the polymer network. In our situation, the rise in the correlation length is due to the stretching and realigning of mucus elastic polymer network by bacteria, a phenomenon similar to that in liquid crystals (5, 6). However, we do not anticipate the onset of long-range liquid crystalline order for the mucin concentrations used in our experiments. Here, the competition between the mucus network’s elastic deformations and the bacteria’s hydrodynamic torques increases spatial correlation length *L*.

The collective bacterial flow has a scale of 80–250 μm. It exceeds the mucin network’s mesh size (2 μm). The recoil during the mesh network distortion can effectively slow the bacterial flow. Besides increasing viscosity, this slowdown may explain the increased temporal correlation in mucin solution with increased concentrations. The Deborah number (De) describes the effect of viscoelastic relaxation of flow patterns. It is defined as $\mathrm{De}=\lambda_{a}/\lambda_{b}$, where $\lambda_{a}$ is the fluid relaxation time and $\lambda_{b}$ is the observation time of fluid flow. Depending on the concentration, the characteristic relaxation time of mucin solution is around $\lambda_{a}∼1-10$ s. The observation time of fluid flow $\lambda_{b}$ associated with the collective bacterial flow is $l_{c}/U_{c}$, where $l_{c}$ is the length of a bacterium and $U_{c}$ is the bacterial flow speed. Thus, $\lambda_{b}$ is ∼0.7–3 s and the Deborah number De∼0.6−14. Therefore, flow elasticity can be essential in the onset of collective motion.

We develop a description of bacterial collective motion in a viscoelastic fluid. It consists of a phenomenological equation for bacterial dynamics (38–40) coupled to the Johnson–Segelman model of polymer viscoelasticity (41, 42). The dynamics of incompressible bacterial liquid is governed by a 2D complex Swift–Hohenberg-type equation for the effective velocity v in thin-film geometry (incorporating bacterial and solvent velocity):


(1) $$\partial_{t}v+v\cdot \nabla v=\nabla \cdot T-\alpha v-\zeta |v{|}^{2}v$$

complemented by the incompressibility condition ∇⋅v=0. Here, T is the stress tensor, the viscous friction term αv is due to surface integration (43), and the nonlinear term $\zeta |v{|}^{2}v$ is needed for saturation of the instability. The “effective” stress tensor T is of the form:


(2) $$T=-pI+2\beta D-2\gamma \nabla^{2}D+\phi (\tau )\Sigma .$$

Here, *p* is the isotropic pressure, I is the 2D identity matrix, Σ is the polymer stress and D is the velocity gradient tensor


(3) $$D=\frac{1}{2}(\nabla v+\nabla v^{T}).$$

Here, the superscript T implies the transpose matrix. Terms $\beta D,\gamma \nabla^{2}D$ include, on the phenomenological level, the active dipolar stresses produced by swimming bacteria, as well as viscous and collision stresses. According to Ref. (30), the increase in viscosity is absorbed in the model coefficients α,β and γ in Eq. 2 and does not change the scale of collective motion. It implies that the effect of increased polymer viscosity, as in PVP360, should modify the coefficients proportionally to a common factor: $\left(\alpha^{\prime },\beta^{\prime },\gamma^{\prime })=(1+g\mu )(\alpha ,\beta ,\gamma \right)$, *g* is phenomenological parameter, *μ* is the polymer viscosity. The phenomenological factor ϕ(τ) characterizes the polymer nonviscous (i.e. elastic) response. Here, *τ* is the polymer relaxation time. Correspondingly, ϕ→0 for τ→0.

The polymer stress Σ is described by the Johnson–Segelman equation:


(4) $$\overset{▽}{\Sigma }=-\frac{1}{\tau }\Sigma +\frac{2\mu }{\tau }D+D_{p}\nabla^{2}D.$$

Here, $\Sigma^{\;▽}$ is the notation for the Gordon-Schowalter time derivative (upper convective derivative), τ is the polymer relaxation time, E=μ/τ is effective polymer elasticity, and $D_{p}$ is the polymer thermal diffusion coefficient (41, 42). For simplicity, we assume polymer deformation small and neglect the polymer nonlinearity, reducing the Gordon–Schowalter upper convective derivative to partial time derivative: $\Sigma^{\;▽}=\partial_{t}\Sigma$. We also neglect the diffusion term $D_{p}\nabla^{2}D$; the coefficient $D_{p}$ is of the order of thermal diffusivity of polymer molecules and is important only for very large deformations (41). The resulting model captures the bacterial collective behavior in Newtonian (for τ=0) and viscoelastic (τ≠0) fluids. It is also significantly simpler than the model for viscoelastic liquid crystals (44, 45).

Thus, resulting equations describing the bacterial collective motion in viscoelastic fluid assume the form


(5) $$\partial_{t}v+v\cdot \nabla v=\phi (\tau )q-\nabla p+(1+g\mu )(-\alpha v+\beta \nabla^{2}v-\gamma \nabla^{4}v)-\zeta |v{|}^{2}v$$

(6) $$\partial_{t}q=-\frac{1}{\tau }q+\frac{\mu }{\tau }\nabla^{2}v,\nabla \cdot v=0.$$

**Fig. 5.**
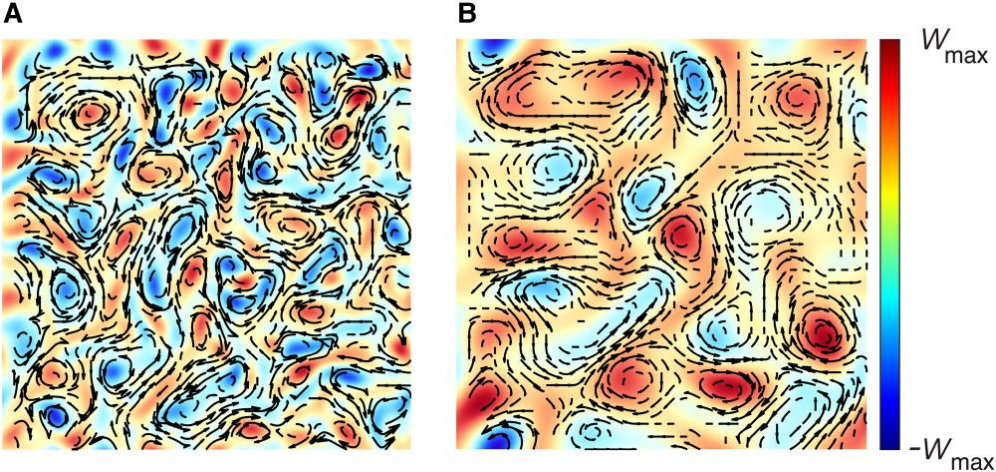
Computational modeling results. A) No polymer stress, ϕ=0. B) Effect of polymer stress, τ=0.4, ϕ=1. Other parameters of the model: domain size 50×50 dimensionless units, β=−1.05, α=0.0303, γ=0.303, ζ=0.01, μ=1.3, g=0.5. The vorticity *w* is shown in pseudocolors, the flow velocity is displayed by arrows.

In the absence of the polymer stress Σ, Eq. 1 exhibits a short-wave instability for β<0 with the optimal wavenumber $k_{0}=\sqrt{|\beta |/2\gamma }$ (38). The instability results in the onset of collective motion closely resembling bacterial turbulence observed experimentally (30, 38, 46). The examination of Eqs. 5, 6 confirmed that the characteristic scale of bacterial collective motion increases with the increase of polymer stress contribution proportional to μϕ(τ)), see Fig. 5 and Supplementary Video 9. Equations 5, 6 also predict the dependence of the characteristic wavelength of the bacterial collective motion $\tilde{k}$ as the following ${\tilde{k}}^{2}=(|\beta |(1+(g-\phi /|\beta |)\tau E)/2(1+g\tau E))$. Here, we used that μ=τE. In the following, we assume g>ϕ/|β|. Then, the increase in the polymer stress will decrease the optimal instability wavenumber and increase the correlation length. Correspondingly, the characteristic length *L* vs. polymer stress behaves as


(7) $$L\approx L_{0}\sqrt{\frac{1+\kappa_{1}E}{1+\kappa_{2}E}},$$

where $L_{0}$ is the characteristic length in the solvent, and $\kappa_{1}>\kappa_{2}>0$ are fit parameters. The dependence agrees with the experimental data in Fig. 4.

## Conclusion

We have shown that the viscoelasticity of suspending media profoundly affects the collective behavior of bacterial active matter. Namely, the elasticity of the solution increases spatial correlation length, whereas the viscosity mainly results in motion slowing down and, in turn, increasing the correlation time. This statement is supported by the correlation measurements in the viscoelastic mucus solution and contrasted with the study of essentially Newtonian PVP360 polymer suspension. We anticipate an onset of a global vortex when the correlation length becomes of the order of system size, similar to (10, 47). However, whether this global vortex exhibits spontaneous direction reversal, as in Ref. (10) could depend on subtle properties of the viscoelastic medium, e.g. the ratio between the relaxation time of the medium and the correlation time of the bacterial locomotion, nonlinearity of the stress–strain relation, etc.

The increased correlation length *L* in mucus with elasticity *E* is the consequence of the reorientation of the mucin network by collectively swimming bacteria. The elastic deformations of mucin network “flattens” small-scale bacterial collective motion and lead to the increased size of bacterial vortices and streams. A similar effect occurs in liquid crystals where the correlation length increases with the elastic constant (5, 6). A corresponding model for mucus requires a set of highly nonlinear equations coupling the local orientation and polymer extension and therefore is challenging. Here, we adopted a simplified framework where the alignment effect described by the polymer’s elastic response factor ϕ(τ)q in Eq. 5. In this approach, the dependence results from pure physical interaction between swimmers and viscoelastic medium; therefore, neither bacteria- nor media-specific. We anticipate that our results provide insights into a broad class of active matter composed of viscoelastic suspending medium (liquid crystals, polymers, biological fluids) and self-propelled agents, both biological (bacteria) and synthetic. The synthetic realizations could include Janus microswimmers energized by chemical reactions, light, or ultrasound (48). Our results also provide insights into bacteria behavior on mucosal surfaces with possible implications for human and animal health. The enhancement of large-scale collective motion of bacteria by mucus viscoelasticity may result in accelerated bacteria penetration and, in turn, faster bacteria colonization of internal tissues.

## Methods

### Bacteria growth and general experimental procedure

Experiments were performed with a DK400 strain of *Bacillus subtilis*. These bacteria have a diameter of 0.8 μm and a body length of 5–7 μm. They were grown on an LB agar plate at room temperature for 2–3 days until scattered colonies were observed. Then, a single colony was inoculated to Terrific Broth fluid (Sigma T5574) in sealed centrifuged tubes under microaerobic conditions. Bacteria grown under microaerobic conditions show enhanced oxygen deprivation resistance and motility at high bacterial concentrations. The bacterial suspension was centrifuged at the end of the log phase corresponding to an optical density of 0.6. The bacterial pellet was mixed with fresh broth, mucin solutions, or PVP360 solutions with different concentrations to produce a bacteria/broth, bacteria/mucin or bacteria/PVP360 mixture. Five microliter of the mixture was pipetted into a 0.25cm×0.25cm square free-standing film. A 10× magnification objective was used to record a 10 s, 30 fps video for each mixture. Each video was processed by a MATLAB PIV script (31). The velocity field obtained from the PIV was used to generate the temporal velocity autocorrelation and spatial velocity correlation functions. Correlation time and length were extrapolated by fitting each correlation function into each exponentially decaying function. For the experiment on individual bacteria movements, one part of the bacterial culture at the log phase was mixed with nine parts of different solutions (mucin or PVP 360) before imaging. The bacteria concentration during imaging (about $10^{7}$ cells/cm${}^{3}$) is much lower than the concentration threshold for the onset of collective motion ($10^{10}$ cells/cm${}^{3}$).

### Synthetic mucus solution and PVP360 solution fabrication

Partially purified mucin powder from the porcine stomach (M1778 Sigma-Aldrich) or PVP360 powder (PVP360 Sigma-Aldrich) was mixed with Terrific Broth (T9179 Sigma-Aldrich) in different ratios to fabricate mucus/PVP360 solutions in different concentrations. Fabricated mucus/PVP360 solution was stored in 4${}^{\circ }$C before the experiments with bacteria and rheological measurements.

### Natural cow cervical mucus

Mucus samples were collected from Holstein dairy heifers during estrus before artificial insemination. All procedures involving animals are reviewed and approved by the Pennsylvania State University Institutional Animal Care and Use Committee (protocol #200346584) and comply with the Guide for the Care and Use of Agricultural Animals in Agricultural Research and Teaching.

The collected mucus was then stored in 50 mL conical tubes and kept at 4${}^{\circ }$C until further use. The mucin content (7.5 mg/mL) of natural mucus was estimated by the weight of lyophilized natural mucus (70 mg) divided by the volume of natural mucus (9.3 mL) while ignoring the weight of other small proteins, lipids, and DNA. Natural mucus was sonicated for 10 min with broth in different ratios and placed in the ice bath for 2 h before experiments. The sonication helps to dilute natural mucus while preventing significant damage to the mucin polymer network. The final mucin concentrations of natural mucus dilutions used in the inset of Fig. 2A are 0.9375 and 1.875 mg/mL. Experiments with natural mucus dilution at a concentration higher than 1.875 mg/mL are unreliable due to the difficulty in preparing homogeneous bacteria/mucus mixture. An example of bacterial collective motion in natural mucus dilution at around 3.75 mg/mL is shown in Supplementary Video 5 illustrating a scale of collective movement at roughly 200 μm.

### Bulk rheology

We observed that mucus oscillation frequency measurements appear to be unreliable due to issues with loading samples, especially for high mucin concentration. Namely, the elastic mucus drop did not fill the rheometer probe uniformly. It leads to a scatter of the experimental data. Nevertheless, the rheometry data show a gradual increase of both storage and elastic moduli with the increase of mucin concentration, Supplementary Fig. S1E and F.

On the contrary, viscous polymer PVP360 solutions show no elasticity as no network is formed. Therefore, instead of using oscillation frequency measurement to probe both viscous and storage modulus as we do with mucus solutions, a flow sweep measurement was used to characterize the viscosity in the PVP360 solutions at different concentrations. Flow sweep measurement was performed on PVP360 solution for small strain values (Supplementary Fig. 1F). Supplementary Fig. S1B and F gives an apparent increase in the average viscosity, which is similar to Ref. (49).

### Microrheology

The viscoelasticity of mucin solutions was also examined by particle tracking microrheology (50). Briefly, the subdiffusion of micron-sized particles (3 μm in diameter) was tracked and extrapolated to obtain the viscous and storage moduli. Supplementary Fig. 1A and B summarizes the frequency-dependent storage (triangles) and viscous (circles) moduli. Overall, both storage and viscous moduli increase as the mucin concentration increases. In Supplementary Fig. S1C and D, the ratio of the viscous modulus to the elastic modulus tan(δ) is presented. Below 150 mg/mL, the viscous modulus dominates (tan(δ)>1) at all frequencies. At high mucin concentrations (150 mg/mL and above), tan(δ) is close to or smaller than 1, indicating a nontrivial elasticity. To probe the microrheology of mucin solutions, we tracked the random, diffusive behavior of micro-sized polystyrene latex particles characterized by the mean square displacement (MSD) *q*, where $q=\langle |r(t)-r_{0}{|}^{2}\rangle$, $r,r_{0}$ are the current and initial positions of the tracer particle, correspondingly. The MSD data of each concentration was converted into frequency-dependent elastic and viscous moduli using a modified algebraic form of the generalized Stokes–Einstein equations:


(8) $$G^{\prime }(\omega )=\frac{G(\omega )}{1+\beta^{\prime }(\omega )}\cos\;\left[\frac{\pi }{2}\alpha^{\prime }(\omega )-\beta^{\prime }(\omega )\alpha^{\prime }(\omega )\left(\frac{\pi }{2}-1\right)\right]$$

(9) $${G^{\prime }}^{\prime }(\omega )=\frac{G(\omega )}{1+\beta^{\prime }(\omega )}\sin\;\left[\frac{\pi }{2}\alpha^{\prime }(\omega )-\beta^{\prime }(\omega )(1-\alpha^{\prime }(\omega ))\left(\frac{\pi }{2}-1\right)\right],$$

where


(10) $$G(\omega )=\frac{k_{B}T}{\pi a\langle \Delta r^{2}(1/\omega )\rangle \Gamma [1+\alpha (\omega )](1+\beta (\omega )/2)}.$$

Here, *a* is the particle radius, $k_{B}$ is the Boltzmann constant, and Γ is the gamma function. α(ω) and β(ω) are the first- and second-order logarithmic time derivatives of the MSD data, respectively, while $\alpha^{\prime }(\omega )$ and $\beta^{\prime }(\omega )$ are the local first- and second-order logarithmic derivatives of G(ω), respectively (50).

### Thermal rotational diffusion coefficient

An estimate for the (thermal) rotational diffusion coefficient (blue and red dashed lines in Fig. 3C) can be found from the Einstein–Stokes relation for an ellipsoid: $D_{R}^{\prime }=3k_{B}T(\log\;(2b/a)-1/2)/8\pi \eta b^{3}$. Here, a,b are the major and minor axis of the ellipsoid-shaped bacteria cell body, η is the effective viscosity of the suspension, $k_{B}$ is the Boltzmann constant, and *T* is the temperature.

### Numerical implementation

To solve Eqs. 5, 6, the following technique was used. First, we introduced a stream function *φ* and applied curl operation to Eq. 5 to exclude pressure *p* and satisfy the incomprehensibility condition. The resulting equation for vorticity $w=-\nabla^{2}\varphi$ was solved by the quasispectral method in periodic boundary conditions (40). Typically, we used 1024×1024 grid points in the domain of the order of 100×100 dimensionless units.

## Supplementary Material

pgad291_Supplementary_Data

## Data Availability

All data are included in the manuscript and/or supporting information.

## References

[1] Bäumler AJ , Sperandio V. 2016. Interactions between the microbiota and pathogenic bacteria in the gut. Nature. 535(7610):85–93. 27383983 10.1038/nature18849 PMC5114849

[2] Aranson I . 2022. Bacterial active matter. Rep Prog Phys. 85:076601. 10.1088/1361-6633/ac723d 35605446

[3] Gompper G , *et al*. 2020 Feb. The 2020 motile active matter roadmap. J Phys Condens Matter. 32(19):193001. 32058979 10.1088/1361-648X/ab6348

[4] Li G , Lauga E, Ardekani AM. 2021. Microswimming in viscoelastic fluids. J Nonnewton Fluid Mech. 297:104655.

[5] Zhou S , Sokolov A, Lavrentovich OD, Aranson IS. 2014. Living liquid crystals. Proc Natl Acad Sci USA. 111(4):1265–1270. 24474746 10.1073/pnas.1321926111 PMC3910648

[6] Genkin MM , Sokolov A, Lavrentovich OD, Aranson IS. 2017. Topological defects in a living nematic ensnare swimming bacteria. Phys Rev X. 7(1):011029.

[7] Figueroa-Morales N , Dominguez-Rubio L, Ott TL, Aranson IS. 2019. Mechanical shear controls bacterial penetration in mucus. Sci Rep. 9(1):1–10. 31273252 10.1038/s41598-019-46085-z PMC6609767

[8] Mirbagheri SA , Fu HC. 2016. *Helicobacter pylori* couples motility and diffusion to actively create a heterogeneous complex medium in gastric mucus. Phys Rev Lett. 116(19):198101. 27232048 10.1103/PhysRevLett.116.198101

[9] Smalyukh II , Butler J, Shrout JD, Parsek MR, Wong GCL. 2008. Elasticity-mediated nematiclike bacterial organization in model extracellular DNA matrix. Phys Rev E. 78(3):030701. 10.1103/PhysRevE.78.030701 18850984

[10] Liu S , Shankar S, Marchetti MC, Wu Y. 2021. Viscoelastic control of spatiotemporal order in bacterial active matter. Nature. 590(7844):80–84. 33536650 10.1038/s41586-020-03168-6

[11] Martinez RM , Wolk DM. 2016. Bloodstream infections. In: Hayden RT, Wolk DM, Carroll KC, Tang Y, editors. *Diagnostic microbiology of the immunocompromised host*. Wiley Online Library. p. 653–689.

[12] Slots J , Slots H. 2011. Bacterial and viral pathogens in saliva: disease relationship and infectious risk. Periodontol 2000. 55(1):48. 21134228 10.1111/j.1600-0757.2010.00361.x PMC7159101

[13] Flemming H-C , Wingender J. 2010. The biofilm matrix. Nat Rev Microbiol. 8(9):623–633. 20676145 10.1038/nrmicro2415

[14] Westerlund B , Korhonen TK. 1993. Bacterial proteins binding to the mammalian extracellular matrix. Mol Microbiol. 9(4):687–694. 7901732 10.1111/j.1365-2958.1993.tb01729.x

[15] Li G , Ardekani AM. 2016. Collective motion of microorganisms in a viscoelastic fluid. Phys Rev Lett. 117(11):118001. 27661719 10.1103/PhysRevLett.117.118001

[16] Bozorgi Y , Underhill PT. 2011. Effect of viscoelasticity on the collective behavior of swimming microorganisms. Phys Rev E. 84(6):061901. 10.1103/PhysRevE.84.061901 22304110

[17] Shen XN , Arratia PE. 2011. Undulatory swimming in viscoelastic fluids. Phys Rev Lett. 106(20):208101. 21668264 10.1103/PhysRevLett.106.208101

[18] Zhu L , Lauga E, Brandt L. 2012. Self-propulsion in viscoelastic fluids: pushers vs. pullers. Phys Fluids. 24(5):051902.

[19] Datt C , Natale G, Hatzikiriakos SG, Elfring GJ. 2017. An active particle in a complex fluid. J Fluid Mech. 823:675–688.

[20] Arratia PE . 2022. Life in complex fluids: swimming in polymers. Phys Rev Fluids. 7(11):110515.

[21] Zöttl A , Yeomans JM. 2019. Enhanced bacterial swimming speeds in macromolecular polymer solutions. Nat Phys. 15(6):554–558.

[22] Bansil R , Turner BS. 2018. The biology of mucus: composition, synthesis and organization. Adv Drug Deliv Rev. 124:3–15. 28970050 10.1016/j.addr.2017.09.023

[23] Lai SK , Wang Y-Y, Wirtz D, Hanes J. 2009. Micro- and macrorheology of mucus. Adv Drug Deliv Rev. 61(2):86–100. 19166889 10.1016/j.addr.2008.09.012 PMC2736374

[24] Davies JM , Viney C. 1998. Water–mucin phases: conditions for mucus liquid crystallinity. Thermochim Acta. 315(1):39–49.

[25] Valeri M , *et al*. 2015. Pathogenic *E. coli* exploits SsLE mucinase activity to translocate through the mucosal barrier and get access to host cells. PLoS ONE. 10(3):e0117486. 10.1371/journal.pone.0117486 PMC4366376 25789808

[26] Katz DF , Morales P, Samuels SJ, Overstreet JW. 1990. Mechanisms of filtration of morphologically abnormal human sperm by cervical mucus. Fertil Steril. 54(3):513–516. 2397794

[27] Overhage J , Bains M, Brazas MD, Hancock REW. 2008. Swarming of *Pseudomonas aeruginosa* is a complex adaptation leading to increased production of virulence factors and antibiotic resistance. J Bacteriol. 190:2671–2679. 18245294 10.1128/JB.01659-07 PMC2293252

[28] Butler MT , Wang Q, Harshey RM. 2010. Cell density and mobility protect swarming bacteria against antibiotics. Proc Natl Acad Sci USA. 107(8):3776–3781. 20133590 10.1073/pnas.0910934107 PMC2840483

[29] Lee A , O’Rourke JL, Barrington PJ, Trust TJ. 1986. Mucus colonization as a determinant of pathogenicity in intestinal infection by Campylobacter jejuni: a mouse cecal model. Infect Immun. 51(2):536–546. 2935499 10.1128/iai.51.2.536-546.1986 PMC262372

[30] Sokolov A , Aranson IS. 2012. Physical properties of collective motion in suspensions of bacteria. Phys Rev Lett. 109(24):248109. 23368392 10.1103/PhysRevLett.109.248109

[31] Thielicke W , Sonntag R. 2021. Particle image velocimetry for MATLAB: accuracy and enhanced algorithms in PiVlab. J Open Res Softw. 9(12):1–14.

[32] Kočevar-Nared J , Kristl J, Šmid Korbar J. 1997. Comparative rheological investigation of crude gastric mucin and natural gastric mucus. Biomaterials. 18(9):677–681. 9151999 10.1016/s0142-9612(96)00180-9

[33] Ran R , Gagnon DA, Morozov A, Arratia PE. 2021. Polymers in swarming bacterial turbulence. arXiv 00068. 10.48550/arXiv.2111.00068, preprint: not peer reviewed.

[34] Martinez VA , *et al*. 2014. Flagellated bacterial motility in polymer solutions. Proc Natl Acad Sci USA. 111(50):17771–17776. 25468981 10.1073/pnas.1415460111 PMC4273371

[35] DiLuzio WR , *et al*. 2005. *Escherichia coli* swim on the right-hand side. Nature. 435(7046):1271–1274. 15988531 10.1038/nature03660

[36] Cao D , Dvoriashyna M, Liu S, Lauga E, Wu Y. 2022. Reduced surface accumulation of swimming bacteria in viscoelastic polymer fluids. Proc Natl Acad Sci USA. 119(45):e2212078119. 10.1073/pnas.2212078119 PMC9659339 36322736

[37] Patteson AE , Gopinath A, Goulian M, Arratia PE. 2015. Running and tumbling with *E. coli* in polymeric solutions. Sci Rep. 5(1):15761. 26507950 10.1038/srep15761 PMC4938119

[38] Wensink HH , *et al*. 2012. Meso-scale turbulence in living fluids. Proc Natl Acad Sci USA. 109(36):14308–14313. 22908244 10.1073/pnas.1202032109 PMC3437854

[39] Dunkel J , *et al*. 2013. Fluid dynamics of bacterial turbulence. Phys Rev Lett. 110(22):228102. 23767750 10.1103/PhysRevLett.110.228102

[40] Reinken H , *et al*. 2020. Organizing bacterial vortex lattices by periodic obstacle arrays. Commun Phys. 3(1):76.

[41] Olmsted PD , Radulescu O, Lu C-YD. 2000. Johnson–Segalman model with a diffusion term in cylindrical Couette flow. J Rheol. 44(2):257–275.

[42] Lu CYD , Olmsted PD, Ball RC. 2000. Effects of nonlocal stress on the determination of shear banding flow. Phys Rev Lett. 84(4):642–645. 11017336 10.1103/PhysRevLett.84.642

[43] Aranson IS , Sokolov A, Kessler JO, Goldstein RE. 2007. Model for dynamical coherence in thin films of self-propelled microorganisms. Phys Rev E. 75(4):040901. 10.1103/PhysRevE.75.040901 17500857

[44] Hemingway EJ , Cates ME, Fielding SM. 2016. Viscoelastic and elastomeric active matter: linear instability and nonlinear dynamics. Phys Rev E. 93(3):032702. 27078422 10.1103/PhysRevE.93.032702

[45] Chi H , Gavrikov A, Berlyand L, Aranson IS. 2022. Interaction of microswimmers in viscoelastic liquid crystals. Commun Phys. 5(1):274.

[46] Sokolov A , Aranson IS, Kessler JO, Goldstein RE. 2007. Concentration dependence of the collective dynamics of swimming bacteria. Phys Rev Lett. 98(15):158102. 17501387 10.1103/PhysRevLett.98.158102

[47] Lushi E , Wioland H, Goldstein RE. 2014. Fluid flows created by swimming bacteria drive self-organization in confined suspensions. Proc Natl Acad Sci USA. 111(27):9733–9738. 24958878 10.1073/pnas.1405698111 PMC4103334

[48] Aranson IS . 2018. Harnessing medium anisotropy to control active matter. Acc Chem Res. 51(12):3023–3030. 30379534 10.1021/acs.accounts.8b00300

[49] Bühler V . 2005. Polyvinylpyrrolidone excipients for pharmaceuticals: povidone, crospovidone and copovidone. Berlin: Springer Science & Business Media, Springer-Verlag.

[50] Dasgupta BR , Weitz DA. 2005. Microrheology of cross-linked polyacrylamide networks. Phys Rev E. 71(2):021504. 10.1103/PhysRevE.71.021504 15783330

