# Supplementary material for: Viscoelasticity enhances collective motion of bacteria
Source: PNAS Nexus. 2023 Sep 6;2(9):pgad291. doi: 10.1093/pnasnexus/pgad291 (PMC10503537; doi:10.1093/pnasnexus/pgad291)
Supplement: pgad291_Supplementary_Data [file pgad291_supplementary_data.zip › PNASNEXUS-PNASNEXUS-2023-00504R-s01.pdf]

Supplementary Information: Viscoelasticity enhances bacteria collective motion

Wentian Liao[1] and Igor S Aranson[1]

[1]Department of Biomedical Engineering, Pennsylvania State University, University Park, 16802, Pennsylvania, USA.

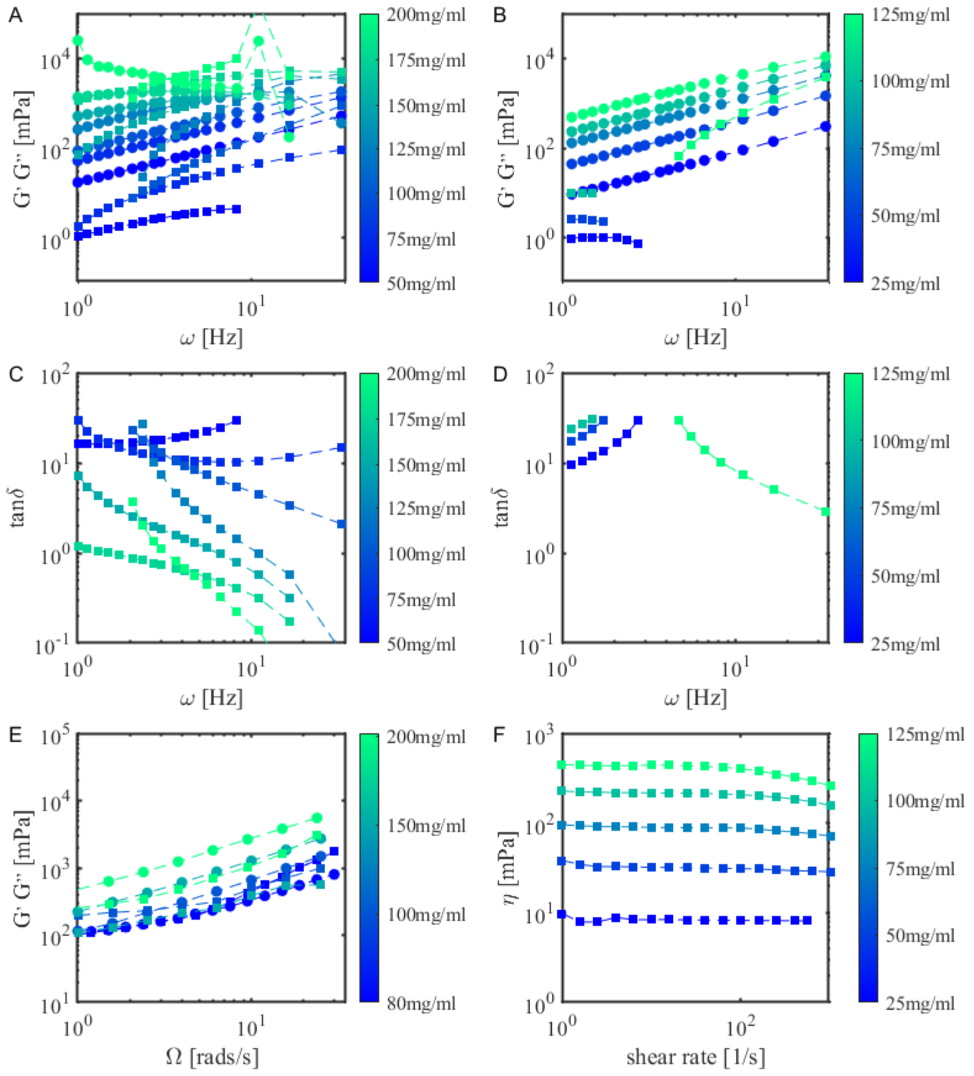

Fig. S1 Rheology of mucin and PVP360 solutions. A. Frequency dependent microrheology of mucin solution. B. Frequency dependent microrheology of PVP360 solution. C. tanδ (G"/G') of mucin solution calculated from Fig .S7.A. D. tanδ (G"/G') of PVP360 solution calculated from Fig .S7.B. E. Oscillation frequency measurement of mucin solution with cone-plate geometry. F. Flow sweep measurement of PVP360 solution with cone-plate geometry.

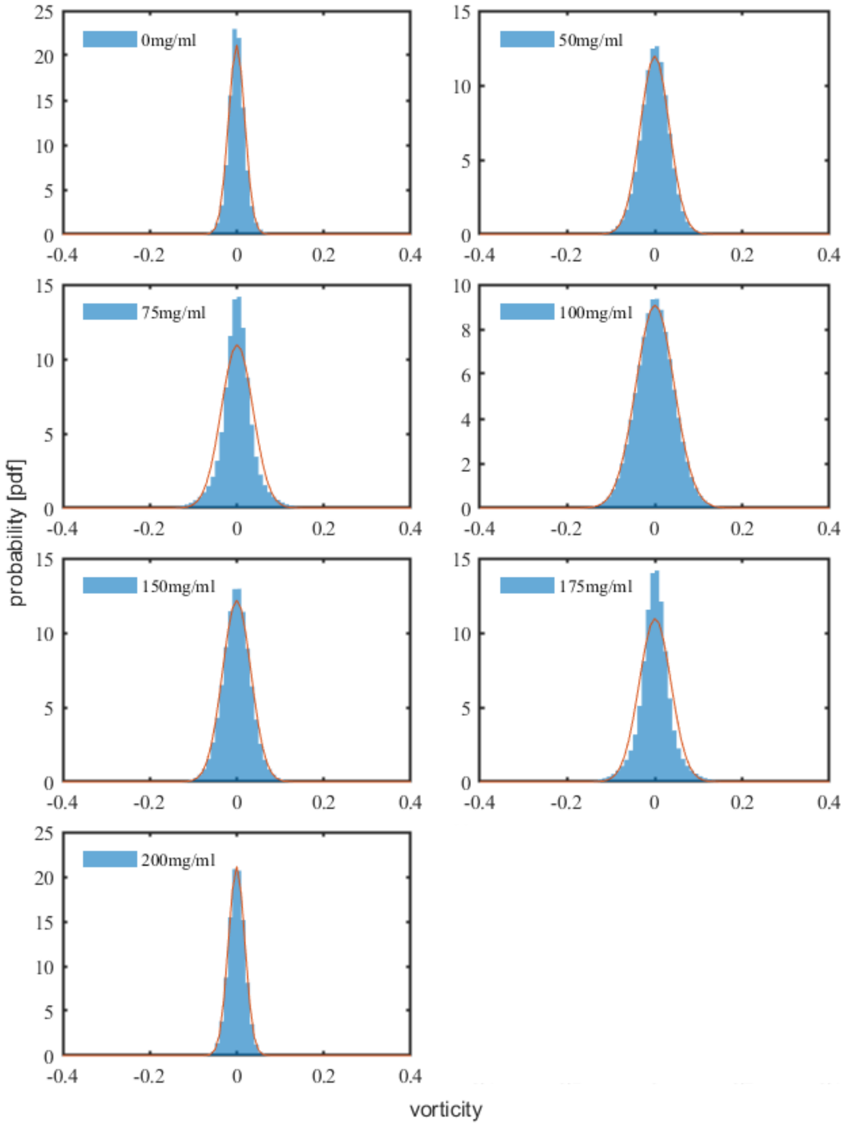

Fig. S2 Vorticity distribution in mucin solutions

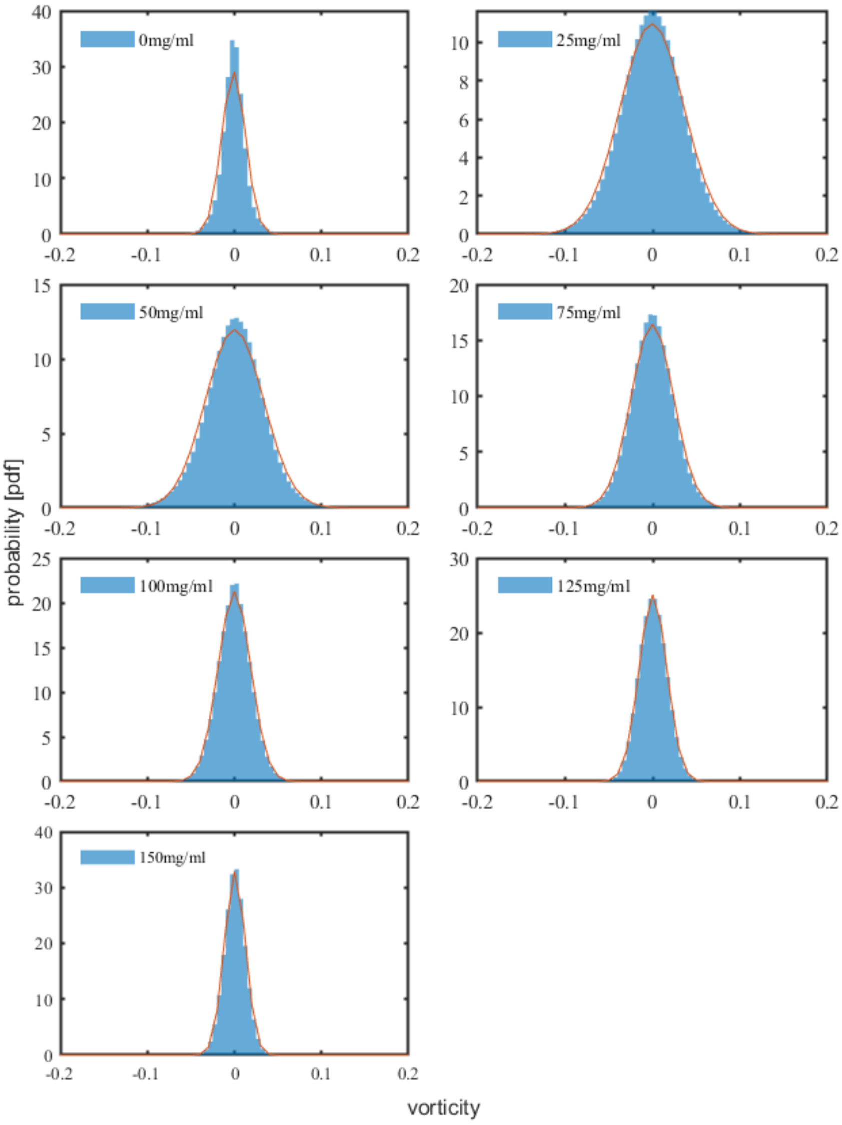

Fig. S3 Vorticity distribution in PVP360 polymer solutions

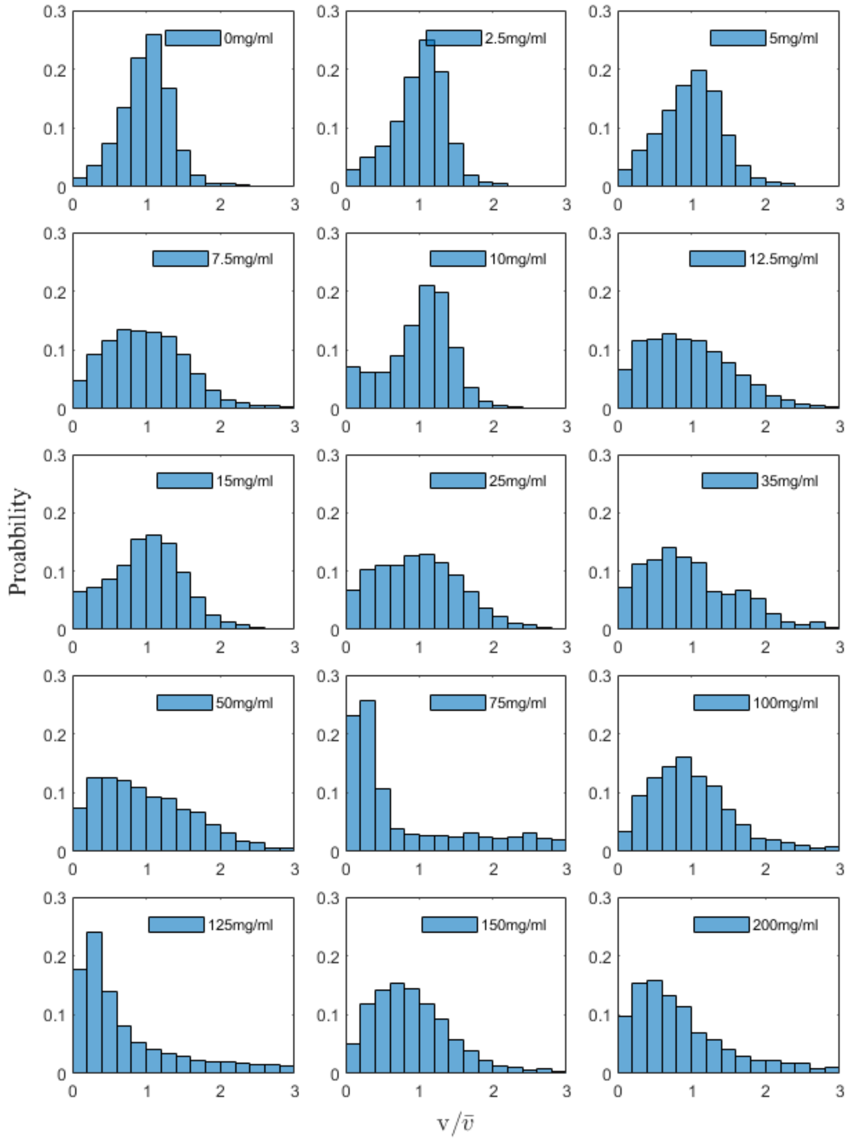

Fig. S4 Velocity distribution normalized by mean speed in mucin solutions

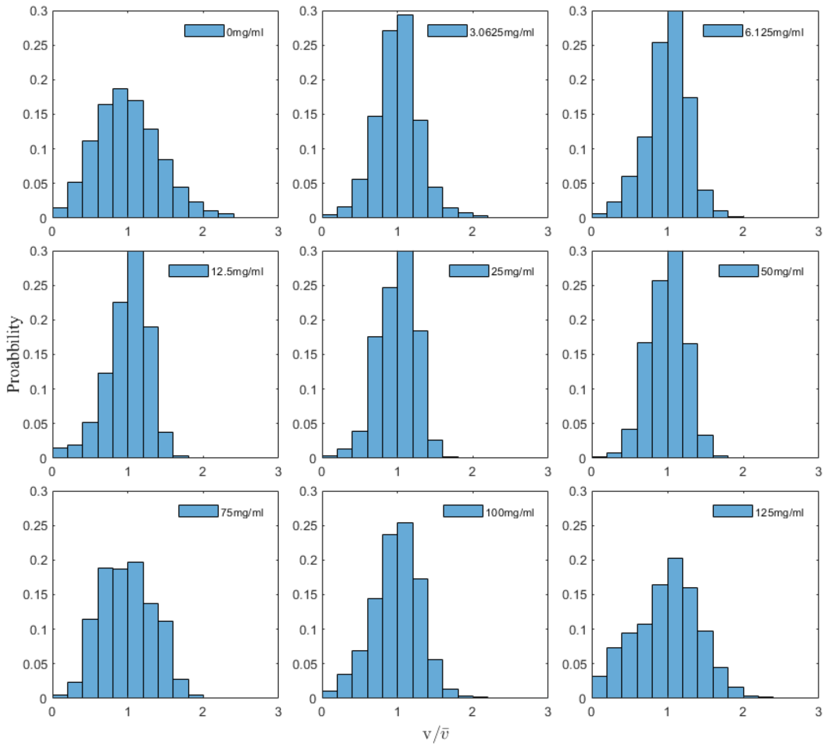

Fig. S5 Velocity distribution normalized by mean speed in PVP360 solutions.png

MSD mucin part1.png

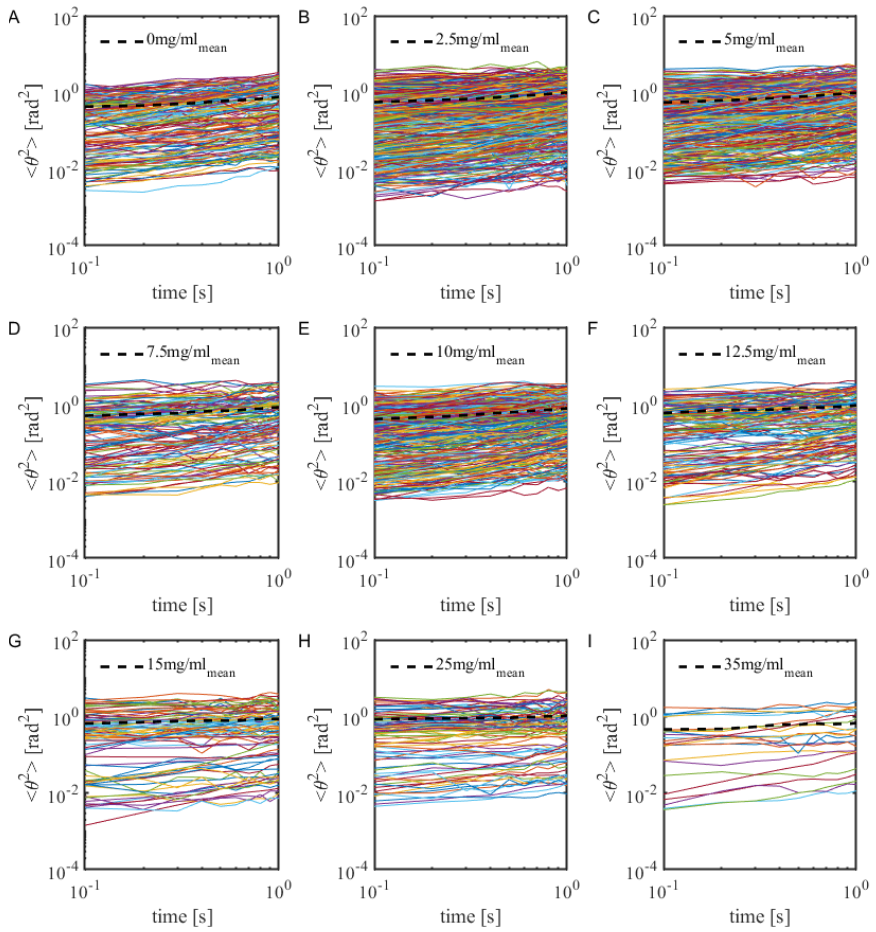

Fig. S6 Angular mean square displacement [rad^2/s] of individual bacterium in mucin solutions

MSD mucin part2.png

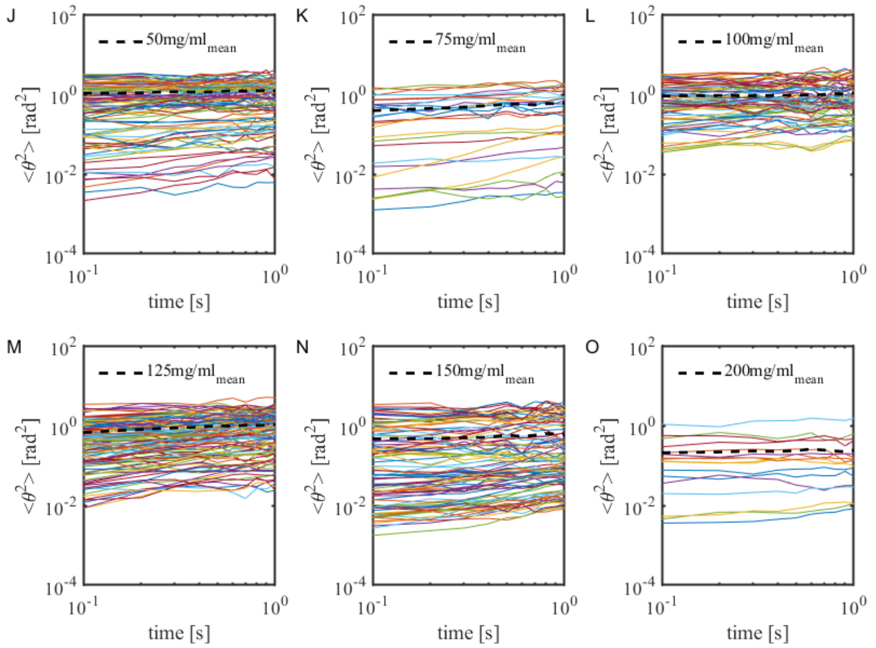

Fig. S7 Angular mean square displacement [rad^2/s] of individual bacterium in mucin solutions

MSD PVP360.png

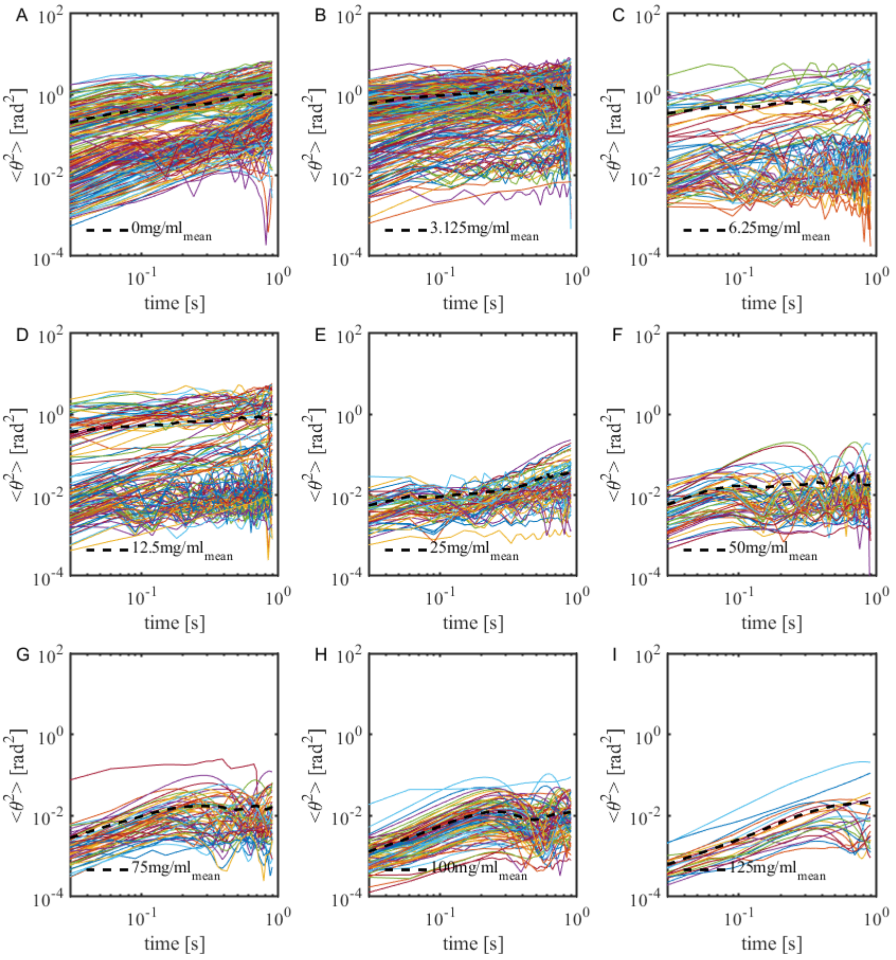

Fig. S8 Angular mean square displacement [rad^2/s] of individual bacterium in mucin solutions

1 Legends to Supplementary Videos

1. Real time flow pattern in 50mg/ml mucin solution.
2. Real time flow pattern in 200mg/ml mucin solution.
3. Real time flow pattern in 25mg/ml PVP360 solution.
4. Real time flow pattern in 125mg/ml PVP360 solution.
5. Real time flow pattern in 3.75mg/ml natural cow cervical mucus.
6. Real time movie of individual bacteria swimming in broth.
7. Three times accelerated movie of individual bacteria swimming in 125mg/ml PVP360 solution.
8. Ten times accelerated movie of individual bacteria swimming in 200mg/ml mucin solution.
9. Computational movie illustrating bacterial dynamics shown in Fig. 5b.
